# Application of machine learning in ophthalmic imaging modalities

**DOI:** 10.1186/s40662-020-00183-6

**Published:** 2020-04-16

**Authors:** Yan Tong, Wei Lu, Yue Yu, Yin Shen

**Affiliations:** 1 Eye Center, Renmin Hospital of Wuhan University, Wuhan, 430060 Hubei China; 2 Medical Research Institute, Wuhan University, Wuhan, Hubei China

**Keywords:** Artificial intelligence, Deep learning, Ophthalmic imaging modalities, Machine learning

## Abstract

In clinical ophthalmology, a variety of image-related diagnostic techniques have begun to offer unprecedented insights into eye diseases based on morphological datasets with millions of data points. Artificial intelligence (AI), inspired by the human multilayered neuronal system, has shown astonishing success within some visual and auditory recognition tasks. In these tasks, AI can analyze digital data in a comprehensive, rapid and non-invasive manner. Bioinformatics has become a focus particularly in the field of medical imaging, where it is driven by enhanced computing power and cloud storage, as well as utilization of novel algorithms and generation of data in massive quantities. Machine learning (ML) is an important branch in the field of AI. The overall potential of ML to automatically pinpoint, identify and grade pathological features in ocular diseases will empower ophthalmologists to provide high-quality diagnosis and facilitate personalized health care in the near future. This review offers perspectives on the origin, development, and applications of ML technology, particularly regarding its applications in ophthalmic imaging modalities.

## Background

Medical imaging is important in clinical diagnosis and individualized treatment of eye diseases [1–3]. This technology can provide high-resolution information regarding anatomic and functional changes. In recent years, imaging techniques have developed rapidly, together with therapeutic advances [4]. However, with the increasing sophistication of imaging technology, comprehension and management of eye disease has become more complex due to the large numbers of images and findings that can be recorded for individual patients, as well as the hypotheses supported by these data. Thus, each patient has become a “big data” challenge [5].

**Fig. 1:**
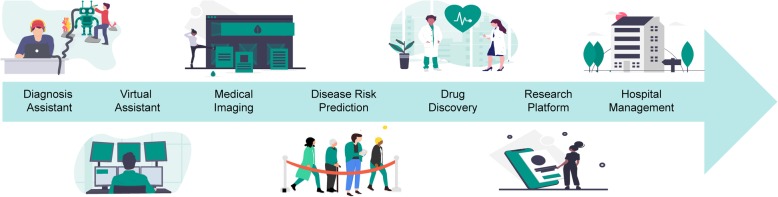
The applications of AI techniques in the eye clinic

Conventional diagnostic methods greatly depend on physicians’ professional experience and knowledge, which can lead to a high rate of misdiagnosis and wastage of medical data [6]. The new era of clinical diagnostics and therapeutics urgently requires intelligent tools to manage medical data safely and efficiently. Artificial intelligence (AI) has been widely applied across various contexts in medicine (Fig. 1). In particular, collaborations between medical imaging and AI disciplines have proven highly productive in the fields of radiology, dermatology and pathology [7].

AI has improved the performance of many challenging tasks in medical imaging, such as diagnosis of cutaneous malignancies using skin photographs [8], detection of lung cancer using chest images [9], prediction of cardiovascular disease risk using computer tomographic (CT) [10], detection of pulmonary embolism using CT angiography [11], analysis of breast histopathology using tissue sections [12], detection of polyps using virtual colonoscopy [13], diagnosis of glioma using magnetic resonance imaging (MRI) [14], and diagnosis of neurological disease using functional MRI (e.g., Alzheimer’s disease) [15–17]. Furthermore, AI has a considerable impact in ophthalmology, mainly through accurate and efficient image interpretation [18].

The rapid increase in AI requires ophthalmologists to embrace intelligent algorithms and gain a greater understanding of the abilities of the technology, and thus enable them to evaluate and apply AI in a constructive manner. Here, we comprehensively reviewed the general applications of ML technology in ophthalmic imaging modalities, including the three most commonly used methods: fundus photography (FP), optical coherence tomography (OCT) and slit-lamp imaging. Throughout the review, we introduce basic definitions of terms commonly used when discussing ML applications, as well as the workflow for building AI models and an overview of the balance between the challenges and opportunities for ML technology in ophthalmic imaging.

## Main text

### From machine learning (ML) to deep learning (DL)

AI refers to the field of computer science that mimics human cognitive function [19]. ML is a subfield of AI that allows computers to learn from a set of data and subsequently make predictions; these processes can be classified as supervised and unsupervised learning.

In supervised learning, a machine is trained with input data previously labeled by humans to predict the desired outcome such that it can solve classification and regression problems. However, this approach is time-consuming because it requires a considerable amount of data to be labeled manually. Conversely, in unsupervised learning, a machine is provided input data that are not explicitly labeled; the machine is then permitted to identify structures and patterns from the set of objects, without human influence. Conventional ML algorithms include decision tree [20], naive Bayes algorithm [21], random forest (RF) [22], support vector machine (SVM) [23, 24], k-nearest neighbor (KNN) [25] (Table 1). Despite obtaining good performance with small datasets, ML network architecture makes them more prone to fail in reaching the convergence and overfitting training dataset because of manual features selection process, which limits their application.

**Table 1:** Representative algorithms in ML and DL

AI Techniques	Classification	Algorithms
Conventional Machine learning	Supervised learning	SVM, Linear Regression, Logistic Regression, RF, KNN, Naïve Bayesian, Decision Tree, AdaBoost, Neural network methods
	Unsupervised learning	Principal component analysis, K-means, Expectation-maximization, Mean shift, Hierarchical clustering, Affinity propagation, Iterative self-organizing data, fuzzy C-means systems
	Reinforcement learning	Q-learning, Temporal difference learning, State-Action-Reward-State-Action, Teaching-Box systems, Maja systems
Deep learning	DBN	Convolutional deep belief network, Conditional restricted Boltzmann machine
	CNN	AlexNet, GoogleNet, Visual geometry group network (VGG), Deep Residual Learning, Inception v4 (v2, v3), Restnet-152 (34,50,101), LeNet
	RNN	Bidirectional RNN, Long short-term memory

*DBN*=deep belief network; *CNN* = convolution neural network; *RNN* = recurrent neural network; *SVM* = support vector machine; *RF* = random forest; *KNN* = k-nearest neighbor

**Fig. 2:**
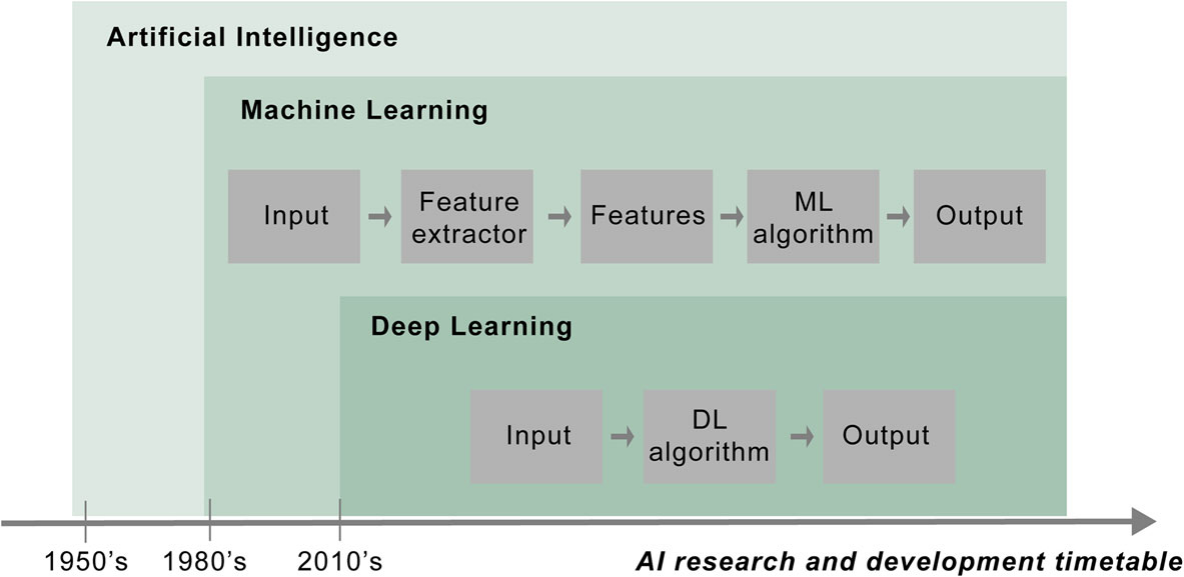
The relationship among the subsets of AI. Machine learning techniques occurred in the 1980s, while deep learning techniques has been applied since the 2010s. Abbreviations: ML, machine learning; DL, deep learning

Among the techniques comprising ML, one of the most promising is DL (Fig. 2) [26]. This mimics the operation of the human brain using multiple layers of artificial neural networks that can generate automated predictions from input data. DL currently has central roles in various tasks, including image recognition (e.g., facial recognition in Facebook, image search in Google), virtual assistant (e.g., Apple’s Siri, Amazon’s Alexa, and Microsoft’s Cortana), and diagnostic assistant systems (e.g. IBM Watson for Oncology). Representative DL algorithms are deep belief network (DBN) [27, 28], convolution neural network (CNN) [29], recurrent neural network (RNN) [30, 31] (Table 1). Compared with conventional ML, the architecture of DL uses more hidden layers to decode image raw data without the need to handcraft specific features or use feature selection algorithm, which has the advantage of efficiency and can explore more complex non-linear pattern in the data (Fig. 2).

**Fig. 3:**
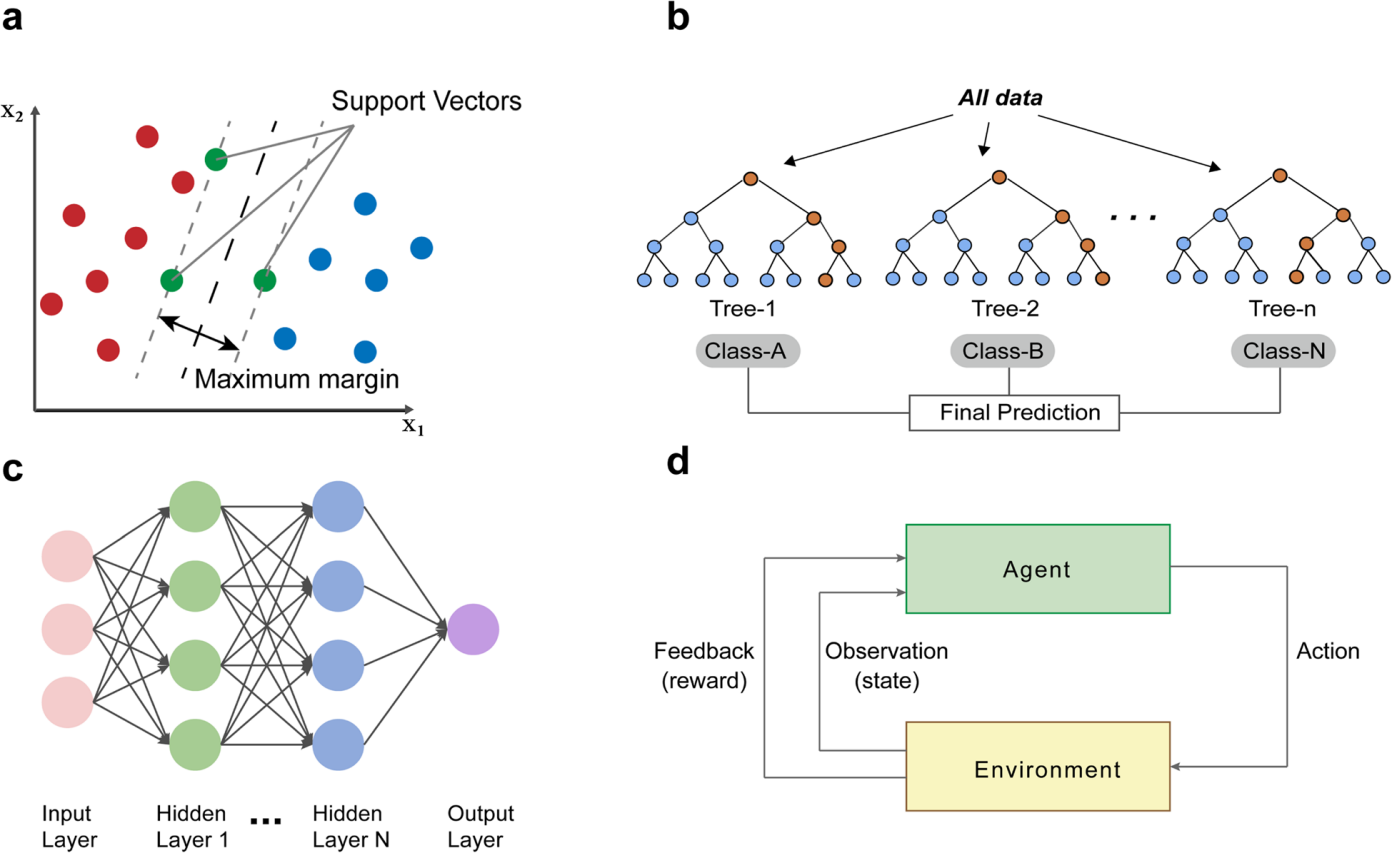
Schematic diagram of common algorithms in AI. **a** SVM are supervised learning models used to analyze the classification and regression of data. **b** RFs are an ensemble learning method that use multiple trees to train and predict samples. **c** CNNs are composed of layers of stacked neurons that can learn complex functions. **d** Reinforcement learning algorithms are used to train the action of an agent on an environment. Abbreviations: SVM, support vector machine; RF, random forest; CNN, convolutional neural networks

**Fig. 4:**
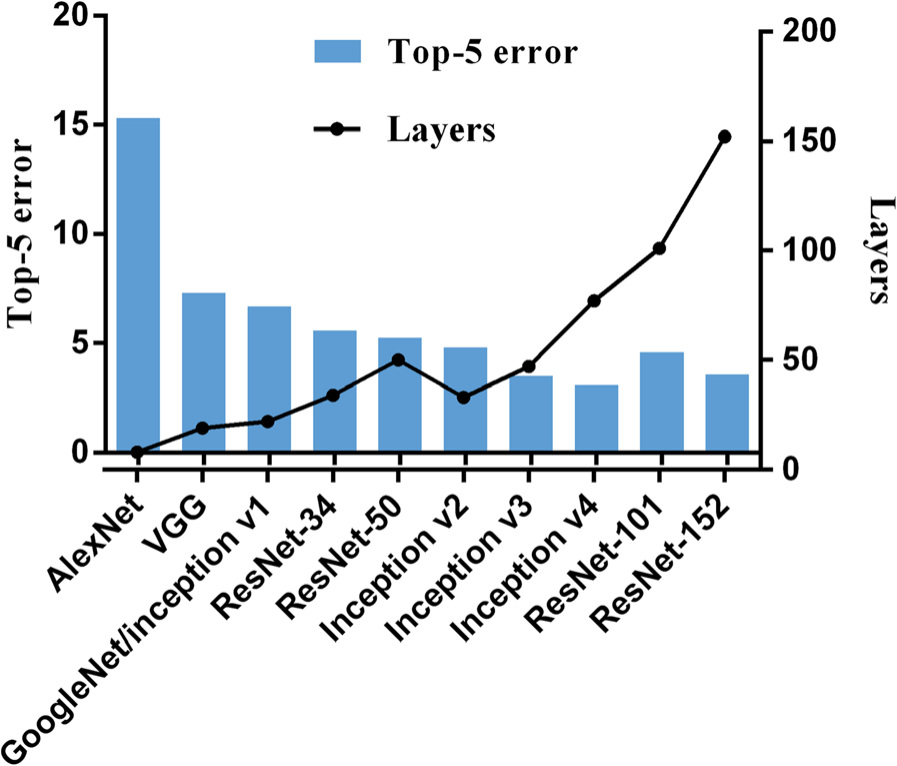
Top-5 error of representative CNN algorithms. Top-5 error: The probability of which none of the first five most probable labels given by the image classification algorithm is correct. Abbreviations: VGG, visual geometry group; GoogleNet, google inception net; ResNet, residual network

**Fig. 5:**
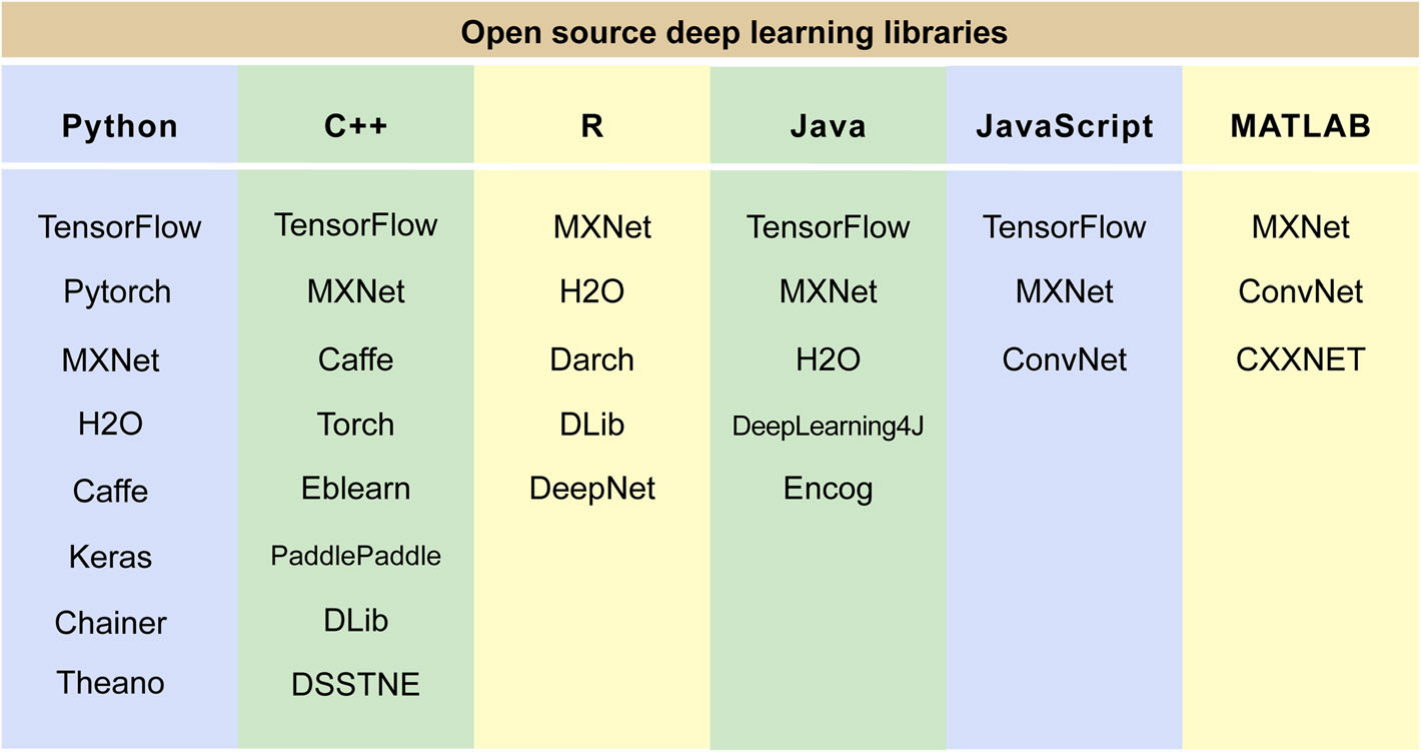
Open source DL research libraries with major programming languages including Python, C++, R, Java. Python libraries tend to be the most popular and can be used to implement recently available algorithms. Abbreviations: DL, deep learning

Visual representation of some common algorithms in ML and DL is shown in Fig. 3. The most commonly applied algorithm in image recognition is CNN. Existing CNN architectures that have been the most widely used include LeNet [32], AlexNet [33], ResNet [34], GoogleNet [35] (Fig. 4), which showed robust performance in the ImageNet Large Scale Visual Recognition Competition [36] and has been successfully applied in facial detection [37], real-time language translation, robot navigation and pedestrian detection [38]. There are various open source tools for development and implementation of AI algorithms; these tools are compatible with many modern programming languages. We summarized some of the most commonly used libraries for DL in Fig. 5.

### AI models building progress

DL neural networks use convolutional parameter layers to learn filters iteratively, which extract hierarchical feature maps from input images, learning the intricate structures of complicated features (such as shapes) through simpler features (such as line) and give the desired classification as output. These convolutional layers are placed in turn, so that each layer transforms the input image and propagates the output information into the next layer.

During the training progress, the parameters (mathematical functions) of the neural network are initially set to random values. The loss function is used to estimate the degree of inconsistency between the predicted value and the true value of the model. Next, the output provided by the function is compared to known features in the training set. Then, parameters of the function are slightly modified by the optimizer so that they can approximate or reach the optimal value, thereby minimizing the loss function. In general, the smaller the loss function, the better the model’s robustness. This process is repeated many times, and the function “learns” how to accurately calculate the features from the pixel intensity of the image for all images in the training set. The most commonly used network is the CNN, which uses a function that first merges nearby pixels into local features and then aggregates them into global features.

**Fig. 6:**
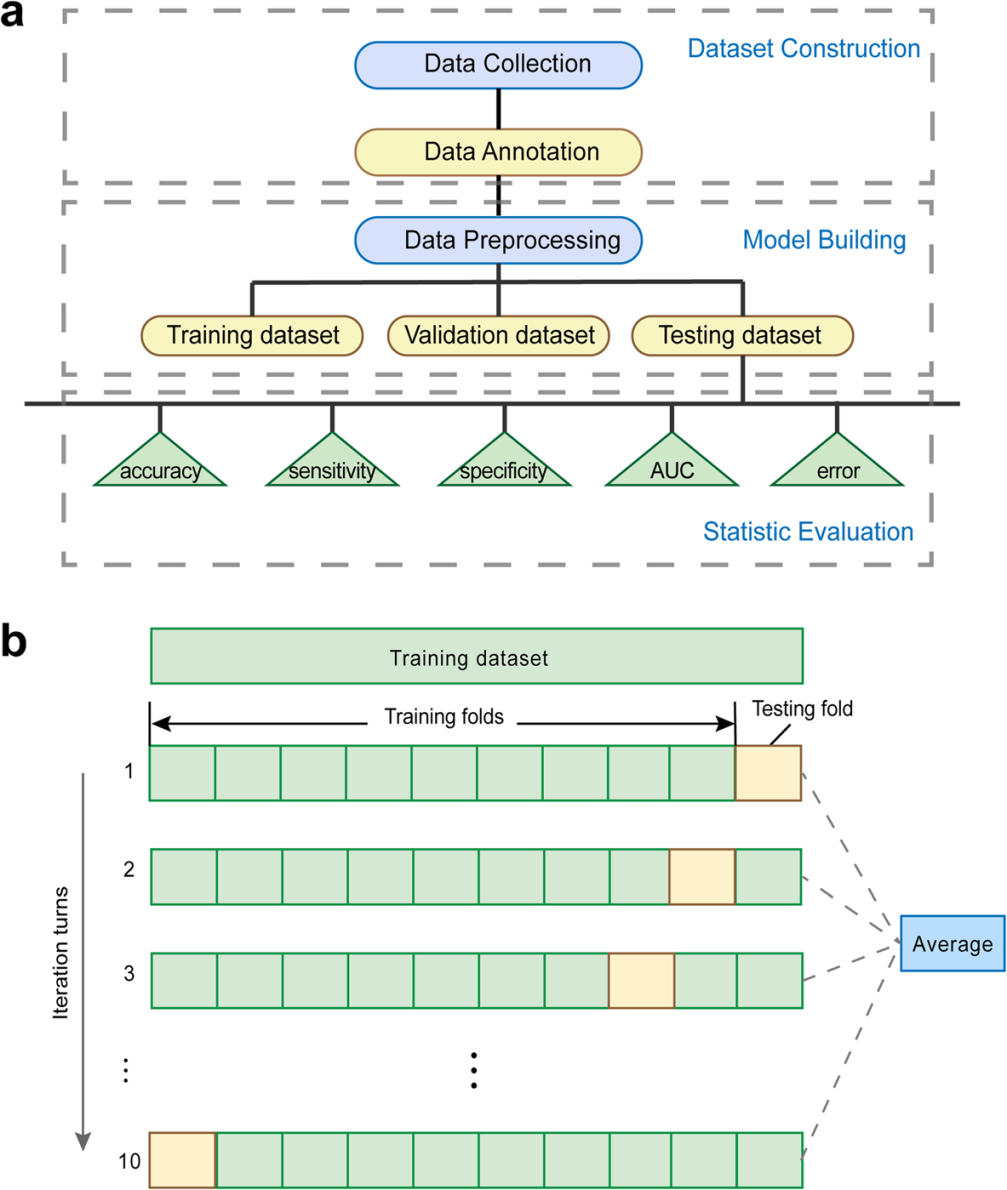
A diagram showing data processing. **a** The typical workflow of AI experimental process. **b** Illustration of k-fold cross-validation techniques (k = 10). Abbreviation: AUC, area under the curve

Figure 6a represents an abstraction of the algorithmic pipeline. The model characterizes the diagnosis of a disease based on an expert-labelled ground truth. The steps for building an AI model include pre-processing image data, training data, validating and testing the model from a large-scale dataset, and eventually evaluate the performance of the trained model.

#### Image data preprocessing

To unify images from different sources and rearrange them into a uniform format, multiple preprocessing steps can be performed [39]: (1) Cleaning up the data: It is the process of reviewing and verifying data, which can remove duplicate information and correct existing errors. (2) Data normalization: The original data will be resized to a common scale which is suitable for comprehensive comparative evaluation. (3) Noise reduction: It will greatly affect the convergence speed of the data and even the accuracy of the trained model if there are a lot of noise in the image data.

#### Training, validation and testing

To achieve a better performance, the base dataset is randomly split into two subsets: one for the model building; and one for testing the model’s performance. The former dataset is further partitioned into training dataset and validation dataset. The training dataset is used to develop the learning model, the validation dataset is used for parameter selection and tuning, and the test dataset was used to evaluate the model.

During the training process, one way to optimize the model and estimate the accuracy of the algorithm when there are insufficient training samples is by using the cross-validation method [40]. All data for modeling is randomly partitioned into k equal sized complementary subsamples. (k-1) folds are selected as the training set and one is selected as the validation set. This process is then repeated across k iterations using a different set of training and testing examples (Fig. 6b).

#### Evaluation metrics

After building the best learning model, evaluation indicators including accuracy, sensitivity and specificity are compared (Table 2). Furthermore, the receiver operating characteristic curve (ROC), and the area under the ROC curve (AUC) indicators are indicative of vital objective evaluation in the task of classification. AUC can measure the accuracies of the positive and negative samples at the same time. The closer the ROC curve is located to upper-left hand corner, the higher the value of AUC, and the better the model’s performance will be.

**Table 2:** Common metrics in AI model evaluation

Evaluation metrics	Definitions
Accuracy	The proportion of both positives and negatives that are correctly identified; the higher the accuracy, the better the classifier
Sensitivity/Recall	The proportion of positives that are correctly identified
Specificity	The proportion of negatives that are correctly identified
Precision	The proportion of positives that are correctly identified among all positive identified samples
Kappa value	To show the actual agreement between two sets of observations
Dice coefficient/F1 score	Harmonic average of the precision and recall, where an F1 score reaches its best value at 1 and worst at 0

### Applications of AI in ophthalmic imaging

**Fig. 7:**
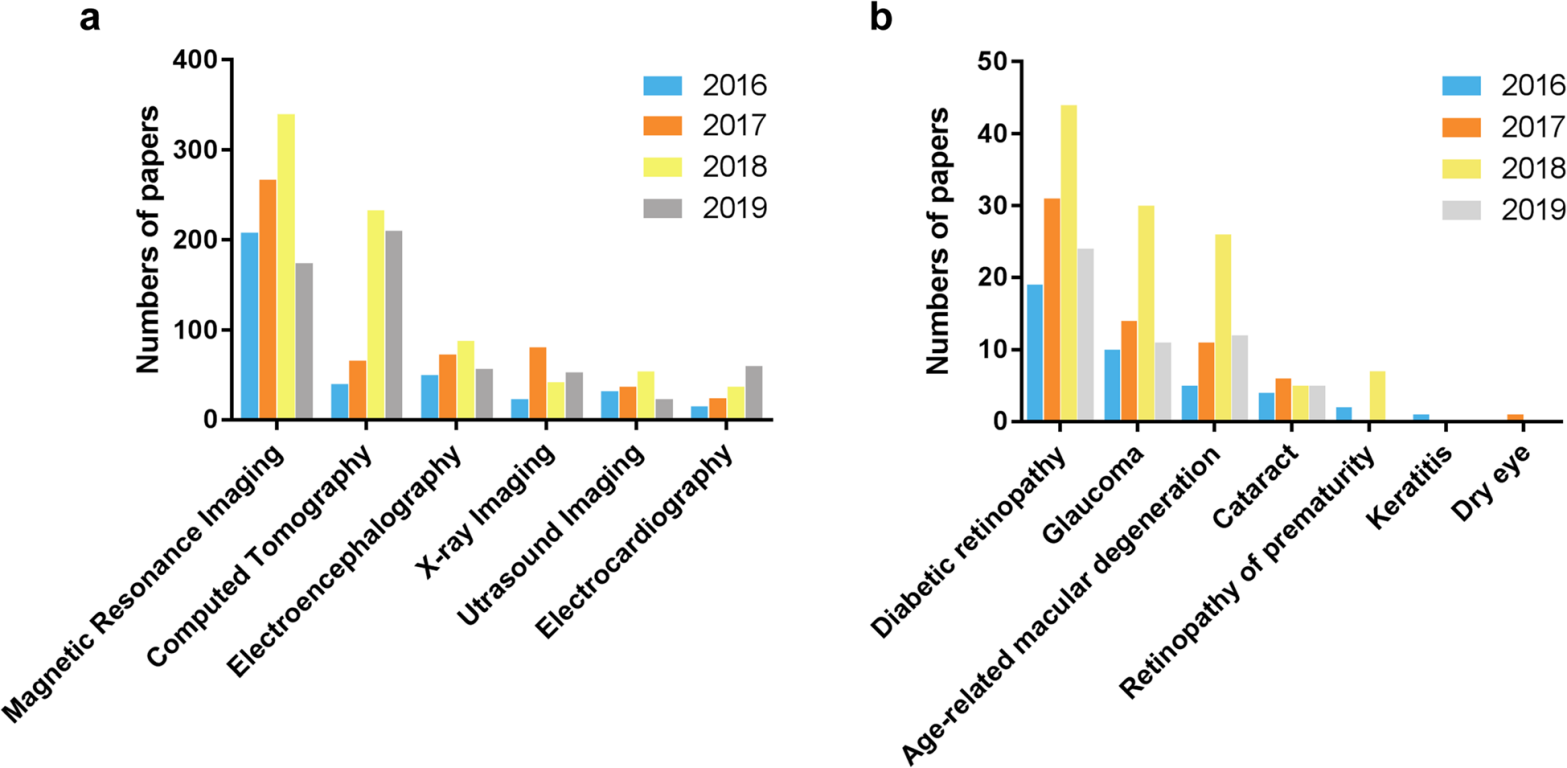
Publication statistics of AI application. **a.** Publication statistics of AI application in different imaging modalities per year indexed on PubMed database (Jan 1st, 2016 to Oct 1st, 2019). **b.** Publication statistics of AI application in diagnosing different ophthalmological diseases per year indexed on PubMed database (Jan 1st, 2016 to Oct 1st, 2019)

Recently, there has been a considerable increase in the use of AI techniques for medical imaging, from processing to interpretation. MRI and CT are collectively used in more than 50% of current articles involving applications of AI in radiology, electroencephalography, electrocardiography, X-ray imaging, ultrasound imaging and angiography (Fig. 7a). Among the applications of AI in ophthalmology, research efforts have focused on diseases with high incidences, such as diabetic retinopathy (DR), glaucoma, age-related macular degeneration (AMD) and cataract (Fig. 7b).

AI may be useful for alleviating clinical workloads as it allows physicians with minimal experience to screen for diseases and detect them in an efficient and objective manner. In the field of ophthalmology, AI has gained increasing interest because it can be used in detecting clinically significant features for diagnostic and prognostic purposes. There have been a number of researches comparing performance between experts and algorithms in diagnosing different ophthalmic imaging modalities.

#### Fundus photograph (FP)

FP is a common ophthalmic imaging technique, in which optical cameras are used to obtain enlarged images of retinal tissues; these retinal photographs are suitable for monitoring, diagnosis, and treatment planning with respect to eye diseases. Various studies have involved the application of AI technology with FP to the diagnosis, grading and monitoring of eye diseases [41, 42].

All diabetic patients need regular retinal screening for early detection and timely treatment of DR [43, 44], which is a leading cause of preventable blindness that affects millions of people worldwide [45]. Specific hallmarks in early DR including exudates [46–48], cotton-wool spots [49, 50], macular edema [51] and micro-aneurysms [52, 53] in the retina can be viewed by FP and identified by AI methods. Most model outputs belong to binary or multi-class classification tasks. Gulshan et al. were the first to use a deep CNN (DCNN) for automated detection of DR [54]. In another study, with a large-scale dataset (494,661 retinal images), a DL system was developed to automatically detect DR, glaucoma, and AMD with respective AUCs of 93.6, 94.2 and 93.1% [55]. Keel and colleagues developed a DL-based DR screening model for use in an endocrinology outpatient clinic, which resulted in 96% patient satisfaction [56].

Generally, conventional FP involves the acquisition of photographs at one-field 45° to the posterior pole of the retina, although the entire retina can be observed at an angle of 230° [57]. Takahashi et al. constructed fundus images of four different shooting directions and trained the GoogleNet DCNN to study single fundus images or four synthetic fundus photos intelligently [58]. The results showed that the accuracy was higher for synthetic fundus images and suggested that wider ranges of fundus images should be used for DR diagnosis. Recently, ultra-wide field scanning laser ophthalmoscopy was introduced; this technology enables scanning of 80% of the fundus area [59]. Diagnosis with wide range FP is an emerging trend in AI diagnostic research, and more advanced algorithms are needed to support its continued growth.

AI can be used in clinical practice to analyze retinal images for disease screening. The Google Chips and Amazon DeepLens cameras, allow embedding of advanced algorithms within devices, which is a useful approach in various medical fields [60]. Rajalakshmi et al. combined an AI-based grading algorithm with a smartphone-based retinal imaging device for potential use in mass retinal screening of people with type 2 diabetes [61]. In 2018, IDx-DR was approved as the first fully autonomous AI-based DR diagnostic system by the United States Food and Drug Administration (FDA) [62]; this study is a milestone as the first prospective assessment of AI in the real-world. We summarized the medical AI products approved by the FDA (Table 3).

**Table 3:** FDA cleared medical AI products

AI products	Production companies	Applications
Kardia App	Kardia Band, Alive Cor, United States	Clinical grade wearable electrocardiogram in Apple Watch
The WAVE Clinical Platform	Excel Medical Electronics, United States	Patient surveillance and predictive algorithm platform
Embrace Watch	Embrace, United States	The smartwatch that uses sensors to measure stress and predict seizures
Viz LVO	Viz.AI, United States	Automatic detection of large vessel occlusion in suspected stroke patients
Cognoa App	Cognoa, United States	An app based on ML that can help clinicians diagnose autism rapidly
Guardian Connect	Medtronic, United States	The continuous glucose monitoring system for people on multiple daily insulin injections
IDx-DR	IDx, United States	To automatic diagnose DR before it causes blindness
OsteoDetect	Imagen Technologies, United States	A type of computer-aided detection and diagnosis software designed to detect wrist fractures in patients
DreaMed Advisor Pro	DreaMed Diabetes, Petah Tikvah, Israel	Automated insulin pump setting adjustments in patients with type 1 diabetes
Viz CTP	Viz.AI, United States	A software package to perform image processing and analysis of CT perfusion scans of the brain

*FDA* = U.S. food and drug administration; *DR* = diabetic retinopathy; *CT* = computer tomographic; *ML* = machine learning

In addition, FP can be used to diagnose other retinal diseases, such as glaucoma, retinopathy of prematurity (ROP), and AMD [63–67]. Recent efforts have aimed to automate pupillary tracking by integrating a motor into the fundus camera. Google Brain has been shown to predict subjects’ cardiovascular risk factors, including age, systolic blood pressure, hemoglobin A1c, and sex from a single fundus image; this task is impossible for professional clinicians [68].

Important issues in the global implementation of ML/DL are the use of big data sharing and open access to scientific data. We have summarized the most commonly used public data-sets of fundus photographs for model training (Table 4). Among them, Kaggle is one of the largest data modeling and data analysis competition platforms in the world, which provides over 50,000 retinal images taken under various shooting conditions, with 0–4 severity level annotated by clinicians. Besides, EyePACS and MESSIDOR are the most commonly used image datasets for DR classification. At present, public eye datasets are mainly applied to automated DR and glaucoma detection, but few for other ophthalmic diseases.

**Table 4:** Common publicly available databases

Datasets	Imaging Modalities	Population	Amount	Annotation
Kaggle	FP	United States	53,576	DR
EyePACS [54]	FP	United States	35,126	DR
MESSIDOR [54]	FP	France	1200	DR; Macular edema
E-OPHTHA [69]	FP	France	463	DR
HRF [70]	FP	Germany	45	DR; Glaucoma; Optic Disk; Vessel;
DRIVE	FP	Netherlands	40	DR; Vessel
RIGA [71]	FP	France; Saudi Arabia	760	Glaucoma
ORIGA-650 [72]	FP	Singapore	650	Glaucoma
DRISHTI-GS [73]	FP	India	101	Glaucoma
INSPRIRE-AVR [74]	FP	United States	40	Glaucoma
REVIEW [75]	FP	United Kingdom	16	Vascular disease

*FP* = fundus photograph; *DR* = diabetic retinopathy

#### Optical coherence tomography (OCT)

OCT is a non-contact and non-invasive optical image-based diagnostic technology, which provides extensive information regarding retinal morphology and assists in the diagnosis of various macular diseases [76]. Thirty million ophthalmic OCT procedures are performed each year; this number is comparable in scale to other medical imaging modalities, such as MRI or CT [77–80]. OCT algorithms can be broadly divided into classification and segmentation tasks.

With appropriate segmentation, the DL algorithm can extract and delineate the structures or lesions in OCT scans, then provide the surface areas or volumes of abnormal regions. Lee et al. applied a CNN model for segmentation of intraretinal fluid in OCT scans, which showed robust performance for interrater reliability between human observers and the algorithm [81]. Another group of patients was assessed regarding the need for urgent referral, using segmentation and classification algorithms. The system could transfer three-dimensional OCT scans into a tissue map and the patients were able to view the video showing the lesion, which sets a new benchmark for future efforts to solve the ‘black box’ problem of neural networks. Notably, the algorithm detected all urgent referral cases within the patient cohort [82]. With the development of DL, some researchers have extended their algorithms to perform segmentation of pigment epithelium detachment, fluid and vessels [83–85].

OCT has become increasingly important in disease detection, prognostication, and surveillance in AMD patients, especially those with wet AMD requiring anti-vascular endothelial growth factor (anti-VEGF). A ML method was proposed to predict the need for anti-VEGF treatment based on OCT scans taken during the intake examination. The results showed that classifications of low- and high-treatment requirement subgroups demonstrated AUCs of 0.7 and 0.77, respectively [86]. Treder et al. showed that a DL algorithm exhibited good performance for automated detection of AMD in spectral domain OCT [87]. This pilot study was an important step toward automated image-guided prediction of treatment intervals in patients with neovascular AMD.

Additionally, OCT can quantitatively measure structural parameters by scanning the thickness of the retinal nerve fiber layer (RNFL), which is recognized as the earliest structure being implicated in glaucoma [88], since the changes are often detectable before visual field loss [89]. ML classifiers have shown substantial diagnostic accuracy for detection of RNFL thickness measurements obtained by OCT [90, 91]. Moreover, algorithms have been developed for the use of OCT parameters to classify the optic disc in patients with open-angle glaucoma [92].

Because DL methods incorporate millions of parameters, the success of these methods largely depends on the availability of large datasets [93]. A DL-based computer-aided system was used to detect DR in a small sample of patients (52 OCT scans), achieving an AUC of 0.98 [94]. Transfer learning is an algorithm that enables the application of cumulative knowledge learned from other datasets to a new task [95]; this algorithm is highly effective with respect to the application of DL, particularly in the context of limited data [63]. An AI diagnostic tool based on a transfer learning algorithm could distinguish OCT images with choroidal neovascularization or diabetic macular edema from those of normal retina with an AUC of 98.9% [96].

Recent research involved analysis of a unique combination of retinal OCT and MRI images; the findings indicated that retinal OCT might provide insights for early diagnosis of neurodegeneration in the brain, including Alzheimer’s disease [97]. Taken together, the results of the above studies highlight the accuracy of diagnostic evaluation using AI.

#### Slit-lamp images

The slit lamp, a high-intensity light source instrument, is used to shine a thin beam of light into the eye, enabling examination of the anterior and posterior segments of the eye. It is applied mainly for wide illumination of much of the eye and its adnexa for general observation.

In recent years, several studies have investigated and made contributions to the grading and classification of senile cataracts by using slit-lamp images. Huang et al. [98] proposed a ranking method based on slit-lamp images and achieved acceptable grading for nuclear cataracts; this could potentially reduce the clinical burden of experienced ophthalmologists. Fan et al. [99] developed an automatic grading system for nuclear sclerosis based on slit-lamp photographs, using linear regression; the grades predicted by that algorithm were statistically reliable. Li et al. [100] extracted important feature landmarks from slit-lamp images and trained an SVM regression model to automatically predict grades of nuclear cataract.

Slit-lamp images are essential in the diagnosis of congenital cataracts, a major cause of childhood blindness [101–103]. Compared with senile cataract, the phenotype of congenital cataract is far more complicated. Slit-lamp images show heterogeneity among cataract patients as well as complexity in their ocular images [104, 105].

In addition, some DL methods for grading and classifying slit-lamp images have shown effective results [106, 107]. Lin and colleagues’ team developed a prototype diagnostic and therapeutic system (CC-Cruiser) for pediatric cataract screening by using preprocessed ocular images and a DCNN [108]; they compared the performances of multiple DL and conventional ML methods from various perspectives [109, 110]. CC-Cruiser has been used in the Ophthalmic Center of Sun Yat-sen University with an accuracy comparable to that of ophthalmologists. Lin and colleagues also built a collaborative cloud-based multihospital AI platform to integrate rare disease data and provide medical suggestions for non-specialized doctors and remote hospitals without advanced equipment. These efforts addressed significant needs in cataract research and may provide a basis for using AI to analyze other ophthalmic images.

With the continual increase in the amount of data available for AI analysis as well as the potential for AI to identify diseases, ophthalmic medical imaging has moved from a strictly conceptual and perceptual approach to more objective methodology. The enhanced efficiency provided by AI is likely to allow ophthalmologists to perform more value-added tasks. In this review, we summarized studies on FP and OCT using DL techniques on diseases with high incidences (Table 5).

**Table 5:** Summary of DL methods using FP and OCT to detect eye disease

Authors	Year	Imaging Modalities	Aim	Data sets	DL techniques	Performance
Arcadu F et al. [111]	2019	FP	Diabetic macular thickening detection	Local:17,997 FPs	Inception-v3	AUC:0.97 (central subfield thickness ≥ 250 μm)0.91 (central foveal thickness ≥ 250 μm)0.94 (central subfield thickness ≥ 400 μm)0.96 (central foveal thickness ≥ 400 μm)
Nagasawa T et al. [112]	2019	FP	Treatment-naïve proliferative diabetic retinopathy detection	Local:132 FPs	VGG-16	Sensitivity: 94.7%Specificity: 97.2%AUC: 0.969
Phan S et al. [113]	2019	FP	Glaucoma detection	Local:3312 FPs	VGG-19ResNet-152DenseNet-201	AUCs of 0.9 or more (3 DCNNs)
Nagasato D et al. [114]	2019	FP	Branch retinal vein occlusion detection	Local:466 FPs	VGG-16SVM	Sensitivity: 94.0%Specificity: 97.0%positive predictive value (PPV): 96.5%negative predictive value (NPV): 93.2%AUC: 97.6%
Burlina PM et al. [115]	2019	FP	To develop DL techniques for synthesizing high-resolution realistic fundus images	Local:133,821 FPs	GAN	AUC:0.9706 (model trained on real data) 0.9235 (model trained on synthetic data)
Girard F et al. [116]	2019	FP	Joint segmentation and classification of retinal arteries and veins	Public:DRIVE, 40 FPsMESSIDOR, 1200 FPs	CNN	Accuracy: 94.8% Sensitivity: 93.7% Specificity: 92.9%
Coyner AS et al. [117]	2018	FP	Image quality assessment of fundus images in ROP	Local: 6043 FPs	VGG-19 DCNN	Accuracy: 89.1% AUC: 0.964
Keel S et al. [118]	2018	FP	Detection of referable diabetic retinopathy and glaucoma	Public:LabelMe, 114,906 FPs (referable DR)		Sensitivity:90% (glaucomatous optic neuropathy) 96% (referable DR)
Sayres R et al. [119]	2018	FP	Assist grading for DR	Public: EyePACS, 1796 FPs	Inception v-4	Sensitivity:79.4% (unassisted) 87.5% (grades only) 88.7% (grades plus heatmap)
Peng Y et al. [120]	2018	FP	Automated classification of AMD severity	Public: AREDS, 59302 FPs	DeepSeeNet (Inception v-3)	Accuracy: 0.671 AUC: 0.94 (large drusen) 0.93 (pigmentary abnormalities) 0.97 (late AMD)
Guo Y et al. [121]	2018	FP	Retinal vessel detection	Public: DRIVE, 20 FPs STARE, 20 FPs	Multiple DCNNs	Accuracy: 95.97% (DRIVE training dataset) 96.13% (DRIVE testing dataset) 95.39% (STARE dataset) AUC: 0,9726 (DRIVE training dataset) 0.9737 (DRIVE testing dataset) 0.9539 (STARE dataset)
Khojasteh P et al. [122]	2018	FP	Detection of exudates, microaneurysms and hemorrhages	Public: DIARETDB1, 75 FPs e-Ophtha, 209 FPs	CNN	Accuracy: 97.3% (DIARETDB1 dataset) 86.6% (e-Ophtha) Sensitivity: 0.96 (exudates) 0.84 (hemorrhages) 0.85 (microaneurysms)
Gargeya R et al. [123]	2017	FP	Automated identification of DR	Public: EyePACS, 75,137 FPs MESSIDOR 2, 1748 E-Ophtha, 463 FPs	DCNN	Sensitivity: 94% Specificity: 98% AUC: 0.97
Burlina PM et al. [63]	2017	FP	Automated grading of AMD	Public: AREDS, more than 130,000 FPs	DCNN	Accuracy: 88.4% (SD, 0.5%)-91.6% (SD, 0.1%) AUC: 0.94 (SD, 0.5%)-0.96 (SD, 0.1%)
Ordóñez PF et al. [124]	2017	FP	To improve the accuracy of microaneurysms detection	Public: Kaggle, 88,702 FPs Messidor, 1200 FPs DiaRerDB1, 89 FPs	Standard CNNVGG CNN	Sensitivity > 91% Specificity > 93% AUC > 93%
Takahashi H et al. [58]	2017	FP	Improving staging of DR	Local: 9939 FPs	GoogleNet DCNN	Prevalence and bias-adjusted Fleiss’kappa (PABAK): 0.64 (modified Davis grading) 0.37 (real prognosis grading)
Abbas Q et al. [125]	2017	FP	Automatic recognition of severity level of DR	Local: 750 FPs	DCNN	Sensitivity: 92.18% Specificity: 94.50% AUC: 0.924
Pfister M et al. [126]	2019	OCT	Automated segmentation of dermal fillers in OCT images	Local: 100 OCT volume data sets	CNN (U-net-like architecture)	Accuracy: 0.9938
Fu H et al. [127]	2019	OCT	Automated angle-closure detection	Local: 4135 anterior segment OCT images	CNN	Sensitivity: 0.79 ± 0.037 Specificity: 0.87 ± 0.009 AUC: 0.90
Masood S et al. [128]	2019	OCT	Automatic choroid layer segmentation from OCT images	Local: 525 OCT images	CNN (Cifar-10 model)	Accuracy: 97%
Dos Santos VA et al. [129]	2019	OCT	Segmentation of cornea OCT scans	Local: 20,160 OCT images	CNN	Accuracy: 99.56%
Asaoka R et al. [130]	2019	OCT	Diagnosis early-onset glaucoma from OCT images	Local: 4316 OCT images	CNN	AUC: 93.7%
Lu W et al. [131]	2018	OCT	Classification of multi-categorical abnormalities from OCT images	Local: 60,407 OCT images	ResNet	Accuracy: 0.959 AUC: 0.984
Schlegl T et al. [132]	2018	OCT	Detection of macular fluid in OCT images	Local: 1200 OCT scans	CNN	Intraretinal cystoid fluid detection: Accuracy: 0.91 AUC: 0.94 Subretinal fluid detection: Accuracy: 0.61 AUC: 0.92
Prahs P et al. [133]	2018	OCT	Evaluation of treatment indication with anti-vascular endothelial growth factor medications	Local: 183,402 OCT scans	GoogleNet inception DCNN	Accuracy: 95.5% Sensitivity: 90.1% Specificity: 96.2% AUC: 0.968
Shah A et al. [134]	2018	OCT	Retinal layer segmentation in OCT images	Local: 3000 OCT scans	CNN	Average computation time: 12.3 s
Chan GCY et al. [135]	2018	OCT	Automated diabetic macular edema classification	Public: Singapore Eye Research Institute, 14,720 OCT scans	AlexNet, VGG, GoogleNet	Accuracy: 93.75%
Muhammad H et al. [136]	2017	OCT	Classification of glaucoma suspects	Local:102 OCT scans	CNN, Random forest	Accuracy: 93.1% (retinal nerve fiber layer)
Lee CS et al. [81]	2017	OCT	Segmentation of macular edema in OCT	Local:1289 OCT images	U-Net CNN	cross-validated Dice coefficient: 0.911
Lee CS et al. [137]	2017	OCT	Classification of normal and AMD OCT images	Public:Electronic medical records, 101,002 OCT images	VGG-16	Accuracy: 87.63% AUC: 92.78%

*DL* = deep learning; *FP* = fundus photography; *OCT* = optical coherence tomography; *CNN =* convolution neural network; *DCNN* = deep convolution neural network; *DR* = diabetic retinopathy; *AMD* = age-related macular degeneration; *AUC* = area under the curve

### Challenges and future considerations

Despite promising findings thus far, there remain challenges and limitations to using AI [138]. First, the quality of input images is inherently variable, primarily because there is a lack of uniform imaging annotation, and there is variability in ocular characteristics among patients. In addition, inter-expert variability in clinical decision making is an important issue which has been well-documented [139]. High inconsistency among experts in the interpretation of ophthalmic images may introduce bias during model training. Secondly, due to the heavy workload of manual annotation, the number of images with clinical annotations is extremely scarce. Hence, advanced image annotation tools should be developed to gather clinical annotations (such as localization of exudates and retinal hemorrhages). Semi-supervised learning method attempts to make full use of unlabeled samples to improve the performance of model generalization. Third, given the complexity of diseases, sufficient data are needed to build high-accuracy models; however, data for more severe stages of disease, as well as for rare diseases, are often insufficient. Fourth, the current application of AI in ophthalmology mainly focuses on single images of a single disease, whereas combined diagnosis using multiple imaging techniques is needed to evaluate diseases in a synergistic manner. Finally, ensuring the security and privacy of medical data is an important challenge that has not been entirely resolved.

In the future, healthcare systems with minimal staff may benefit from modern automated imaging. The inclusion of intelligence within ophthalmic devices may enable healthcare professionals to provide better patient care. Furthermore, AI systems may be embedded within ophthalmic imaging devices for real-time image diagnosis (e.g., portable fundus cameras and smartphones) with minimal operator expertise. Emerging multimodal imaging techniques, which coincide with improved intelligent algorithms, enable joint training from complementary modalities that have different strengths. This embedded AI will be enabled by improved hardware performance with decreasing cost. With the increasing employment of AI in medical care, patients could be self-screened without supervision before an ophthalmologist appointment. Besides, patients in remote areas could receive routine eye examinations and undergo monitoring of disease progression without the intervention of highly skilled operators. Increasing the interpretability of networks will be another important research direction. The “black box” problem has been identified as an obstacle to the application of DL in healthcare. Existing studies have developed novel algorithms that enable clinicians to inspect and visualize the decision process (e.g., OCT tissue-segmentation), rather than simply obtaining a diagnosis suggestion [82]. In terms of treatment, the research on ophthalmic robots needs further exploration; there have been studies on robotic intraretinal vascular injection and anterior macular surgery.

## Conclusions

With the unprecedented progress of computer and imaging technologies, medical imaging has developed from an auxiliary examination to the most important method for clinical and differential diagnosis in modern medicine. High-accuracy models suggest that ML can effectively learn from increasingly complicated images with a high degree of generalization, using a relatively small repository of data [68]. To some extent, AI may revolutionize disease diagnosis and management by performing classifications of difficult images for clinical experts, as well as by rapidly reviewing large amounts of images. Compared with evaluations by humans, AI has advantages in terms of information integration, data processing, and diagnostic speed. Most AI-based applications in medicine are still in early stages; AI in medical care may ultimately aid in expediting the diagnosis and referral of ophthalmic diseases through cross-disciplinary collaborations of clinicians, engineers, and designers.

## Data Availability

Not applicable.
